# Design of Multi-Wavelength Optical Sensor Module for Depth-Dependent Photoplethysmography

**DOI:** 10.3390/s19245441

**Published:** 2019-12-10

**Authors:** Sangjin Han, Donggeun Roh, Junyung Park, Hangsik Shin

**Affiliations:** Department of Biomedical engineering, Chonnam National University, Yeosu 59626, Korea; tkdwls9045@gmail.com (S.H.); nodg0426@gmail.com (D.R.); junyung.pak@gmail.com (J.P.)

**Keywords:** blood volume, light penetration, multiple wavelength, optical sensor, photoplethysmography

## Abstract

The multi-wavelength photoplethysmography sensors were introduced to measure depth-dependent blood volume based on that concept that the longer the light wavelength, the deeper the penetration depth near visible spectrum band. In this study, we propose an omnidirectional optical sensor module that can measure photoplethysmogram while using multiple wavelengths, and describe implementation detail. The developed sensor is manufactured by making a hole in a metal plate and mounting an LED therein, and it has four wavelength LEDs of blue (460 nm), green (530 nm), red (660 nm), and IR (940 nm), being arranged concentrically around a photodetector. Irradiation light intensity was measured by photoluminescent test, and photoplethymogram was measured with each wavelength simultaneously at a periphery of the human body such as fingertip, earlobe, toe, forehead, and wrist, in order to evaluate the developed sensor. As a result, the developed sensor module showed a linear increase of irradiating light intensity according to the number of LEDs increases, and pulsatile waveforms were observed at all four wavelengths in all measuring sites.

## 1. Introduction

**Figure 1:**
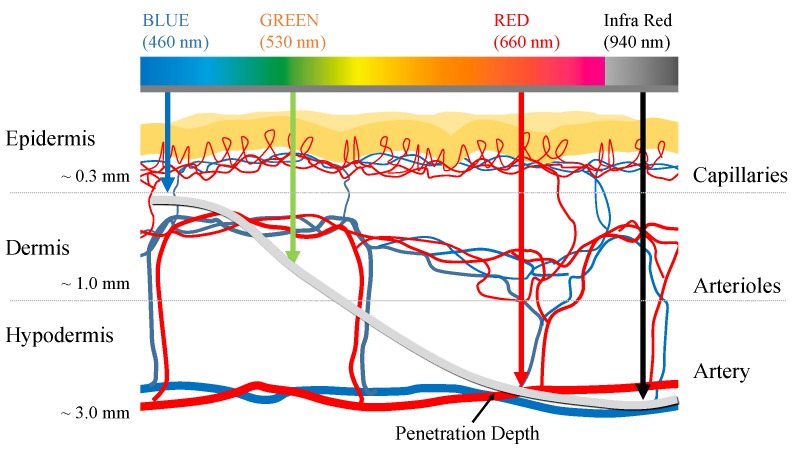
Mean light penetration depth according to the wavelength. Penetration depth according to the light wavelength is referenced from [10].

Photoplethysmography (PPG) is a non-invasive technique for optically measuring the blood volume changes in the vascular bed under the skin [1]. PPG mainly uses light sources of red or infrared wavelengths and it estimates changes in blood volume based on the light absorption characteristics of hemoglobin. For example, as the blood volume of the microvascular bed increases due to cardiac contraction, the amount of light absorbed increases as the number of hemoglobin increases, and the amount of light that passes through the measurement site, such as the finger, decreases. On the contrary, as the blood volume of the microvascular bed decreases due to cardiac dilation, the amount of light absorbed decreases, and the amount of transmitted light increases as the concentration of hemoglobin in the measurement site decreases. As a result, rising and falling are observed in PPG waveform according to the heart beating, which is called a pulsatile component. The PPG sensor consists of a light source and a photodetector (PD). The intensity of light is attenuated as light from the light source passes through the skin due to the absorbance depending on concentration of absorbers and optical path length of the medium according to the Beer–Lambert law [2,3]. Afterwards, the amount of light that passes through the skin is measured by the photodetector. The information obtained using PPG can differ according to the penetration depth of light at particular wavelengths. Generally, it is known that the light penetration depth deepens as the wavelength increases [4]. In recent years, multi-wavelength PPG has been proposed for analyzing pulse waves in blood vessels of different depths using light sources of various wavelengths [5,6,7,8,9,10]. The most representative application using multi-wavelength PPG is blood pressure estimation that is based on local pulse wave velocity. There are studies to measure local pulse wave velocity in microvascular structure and apply it to blood pressure estimation from the premise that pulse wave velocity is related to blood pressure [5,6,7,8]. In general, it is known that wavelengths in the range of 390−600 nm penetrate to the superficial tissue, and longer wavelengths in the range of 600−1100 nm, which penetrate further (Figure 1) [11]. Based on these characteristics, attempts have been made to obtain PPG measurements from various blood vessel depths while using different wavelength light sources. Previous research by Spigulis et al., and Liu et al., demonstrated the feasibility of multi-wavelength PPG for measuring arterial blood volume changes at different depths [5,6,7,8,9,10]. However, the sensors that were proposed in the previous studies require a complicated setup, including an optical fiber and spectrometer [9,10], thus it has a limitation in portable or daily purposes. In other words, a sensor module having a simpler structure is required in order to be used as a wrist watch or band form factor, which is frequently used for health care currently. Moreover, most of existing sensors had non-uniform distances between the light sources of each wavelength and the photodetector [5,6,7,8]. Further, since the light source was only located in one direction of the photodetector, it could not reflect the blood volume change in a common vascular bed while considering the topological and geometric irregularities of a microvascular network [12], which is a significant limitation. Therefore, we developed an omnidirectional multi-wavelength PPG sensor in order to improve these constraints. Previous studies have shown that placing multiple LEDs around a photodetector can improve the quality of the PPG [13]; moreover, the use of multiple light sources is also common in wrist watch type pulse monitors that are already commercially available. However, there is still little research on the sensor structure for depth-dependent PPG measurement. The proposed sensor has a structure in which a plurality of LEDs is arranged in a circle around a photodetector. The new sensor has the potential to provide a solution to the problems of directionality between the light source and the photodetector, the non-uniform distance between the light source and the photodetector, and the light amount control function.

## 2. Omnidirectional Multi-wavelength PPG sensor

### 2.1. Sensor Structure and LED Circuit

**Figure 2:**
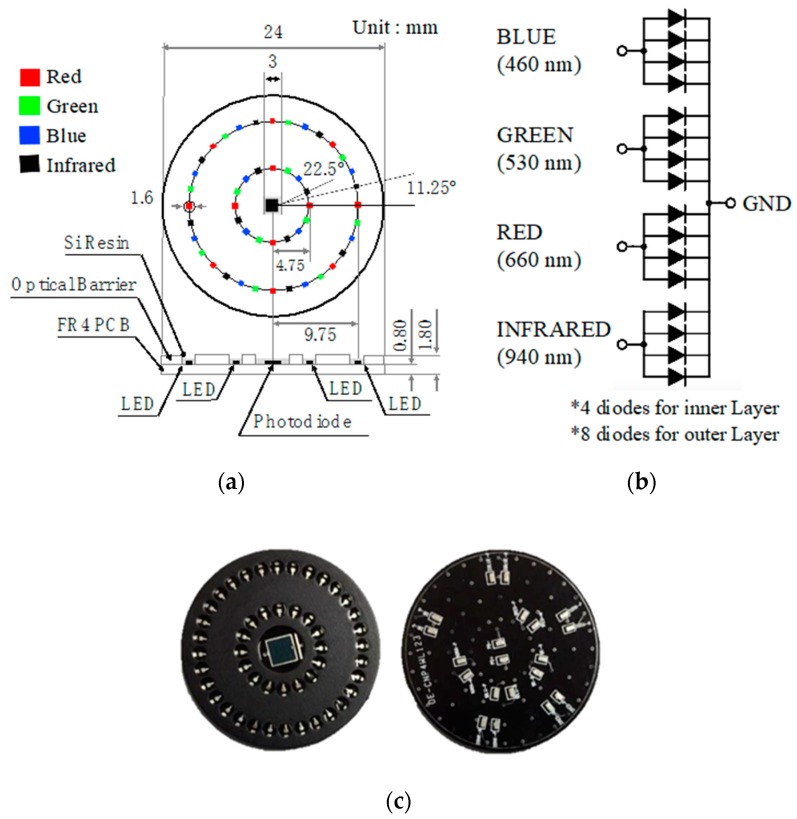
Proposed multi-wavelength photoplethysmography (PPG) sensor, (**a**) structure and dimension, (**b**) LED circuit, and (**c**) front- and back-side of the fabricated sensor.

**Figure 3:**
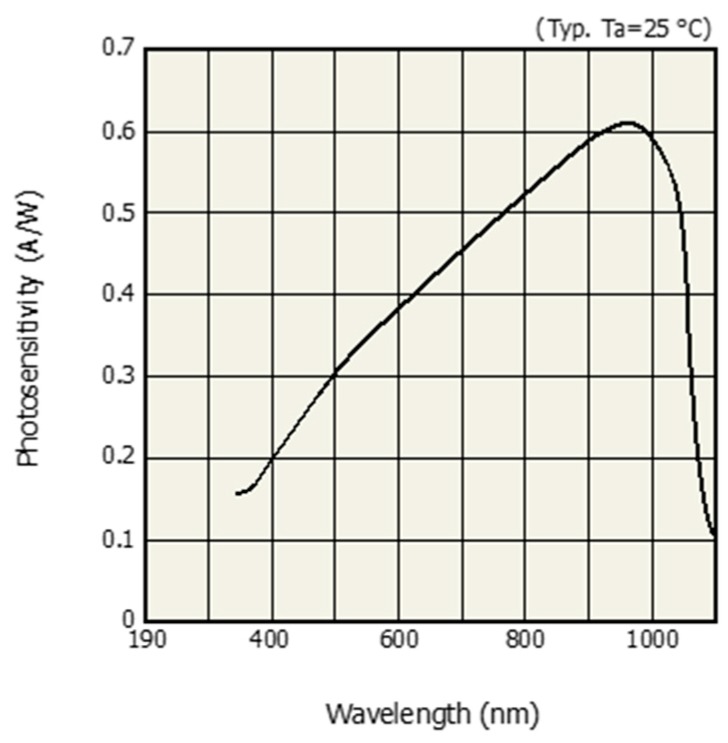
Spectral sensitivity of photodetector [14].

The multi-wavelength optical sensor was designed so that multiple LEDs were arranged in a circle around a photodetector. Blue (460 nm), green (530 nm), red (660 nm), and IR (940 nm) LEDs were used as the light sources and the LEDs were placed on two concentric circles with radii of 4.75 mm and 9.75 mm, respectively. Radii of 4.75 mm and 9.75 mm are the minimum radii for omnidirectional placing for 16 LEDs and 32 LEDs, respectively. The dual column configuration—the outer row and the inner row—are intended to examine the effect of distance-light on PPG measurements. The light sources were arranged so that the respective wavelengths were sequentially repeated. Each LED was arranged at an interval of 11.25 degrees. A total of 16 LEDs was mounted on the inner layer, four for each wavelength, and a total of 32 LEDs are mounted on the outer layer, eight for each wavelength. The developed optical sensor had a diameter of 24 mm and a thickness of 1.8 mm. The surface of the printed circuit board of the sensor was treated with black to prevent light reflection. In addition, a 1 mm high spring steel light barrier was stacked on the top of the printed circuit board (PCB) to prevent light that is emitted from the light source from directly reaching the PD, without passing through the skin. The light barrier at the spot where the individual LEDs were located was processed into a hole pocket to reveal the LED. The exposed LED and PD chip were protected with silicone resin. Figure 2a shows the dimension and structure of the developed sensor and Figure 2b shows the internal circuit of the developed sensor. Figure 2b shows how the BLUE, GREEN, RED, and INFRARED ports were connected to the analog front-end (AFE) for optical application. In this study, we used two AFE4404s (Texas Instruments, Inc., Dallas, TX, USA) as the optical AFEs to measure the four PPG wavelengths and ambient light intensity. Figure 2c shows the developed omnidirectional multi-wavelength optical sensor and Figure 3 shows the spectral sensitivity of photodetector of developed sensor [14].

### 2.2. Photopluminescence Test

**Figure 4:**
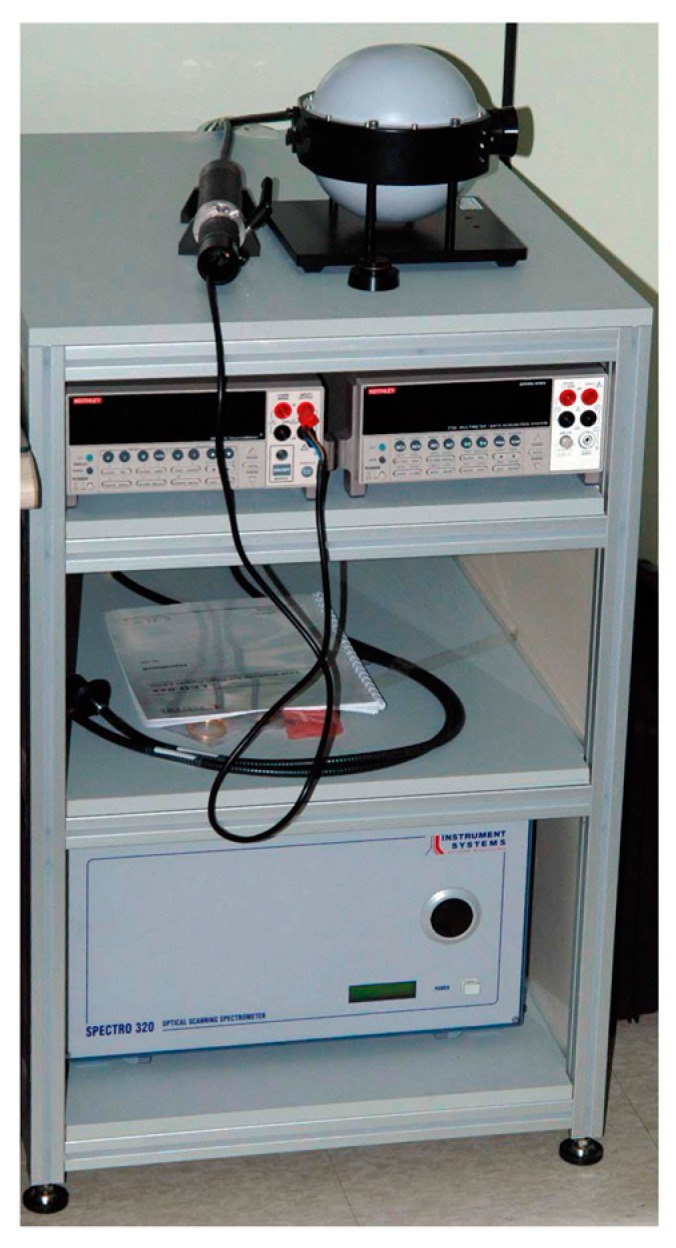
Configuration of the spectrometer system.

The fabricated multiwavelength optical sensor was designed to change the number of LEDs and input current by each wavelength. The photoluminescence test was carried out by changing the number of LEDs and the driving current for each wavelength in order to verify the operation of the multiwavelength optical sensor. The number of LEDs was changed to 1, 2, 4, and 8, and the input currents were changed to 6, 9, 12, 15 and 18 mA. Photoluminescence performance was evaluated by radiometry. An LED integrating sphere, ISP 150, spectrometer, Spectro 320 Scanning Spectrometer +2420 (Instrument Systems, Munich, Germany) were used as the photometry and radiometry devices. Photometry was used to measure the luminous flux of the lumen unit and radiometry measured the power (W). Figure 4 shows the spectrometer system configuration and Table 1 shows the specifications of the Spectro 320 Scanning Spectrometer. The measurements were repeated three times under the same conditions and the mean values were obtained. The maximum measurable wavelength for the SP320 used in the photoluminescence test was 920 nm and the wavelength of the infrared LED applied to the proposed sensor was 940 nm. The infrared light source was excluded from the test.

### 2.3. Pulse Measurement Test

**Figure 5:**
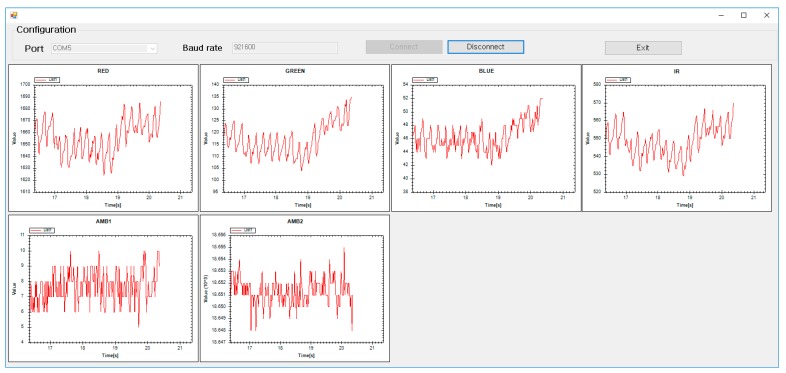
Computer application used to record the multi-channel PPG signal.

The ultimate goal of this study was to measure pulsation waveforms. Therefore, the blood volume change was measured at a peripheral site of the human body, such as fingertip, earlobe, and toe, where the skin thickness was relatively thin and the vascular bed was developed. In addition, the developed sensor was used to measure the position of the wrist, forehead, etc., where PPG could be used effectively. The blood volume change at the end of the human body was measured by transmission, where the amount of blood was estimated according to the amount of light absorbed while the irradiated light passed through the human body and, by reflection, where the volume was estimated by measuring the light intensity returned from scattering and the reflection of transmitted light. We obtained multi-wavelength PPG signals from the fingertip while using the developed sensor to verify the operation of the developed sensor. The signals were acquired at a sampling frequency of 500 Hz for each wavelength through RS232C serial communication and the signal was acquired while using a C#-based in-house application shown in Figure 5. A total of six channel signals, including four PPGs that were measured in red, green, blue, and infrared wavelengths, and ambient light from two AFEs were simultaneously recorded using the above computer application for post-processing of PPG waveform.

## 3. Results and Discussion

### 3.1. Photopluminescence Test

**Figure 6:**
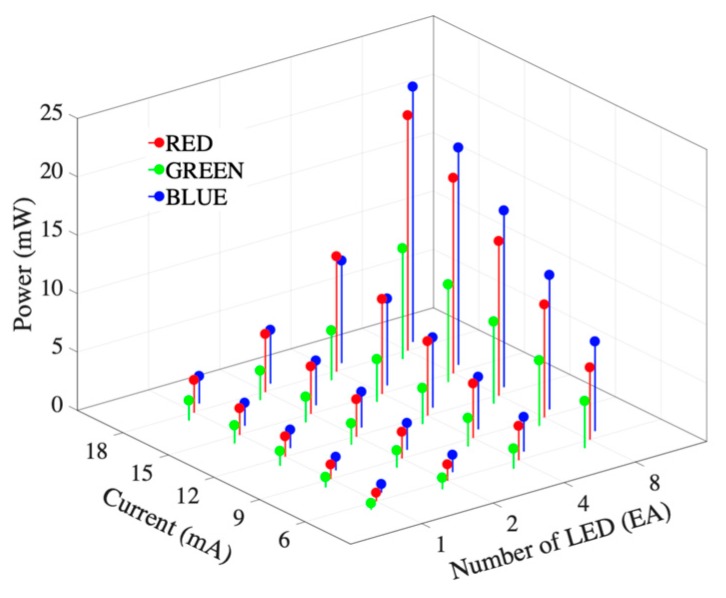
Results of the radiometry test of the developed sensor.

**Figure 7:**
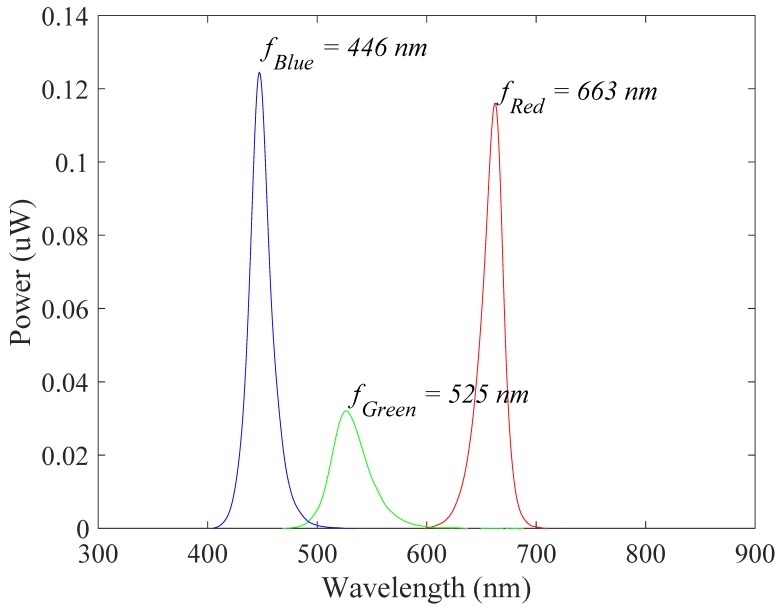
Spectral characteristic and dominant frequency of LEDs according to the photoluminescence test.

Figure 6 shows the results of the radiometry tests of the developed sensor. The number of LEDs and the driving current intensity increase the radiated power. The emitted power was relatively low at the green wavelength and similar for the red and blue wavelengths. Table 2 shows the radiometric results for each wavelength according to the applied current and number of LEDs. In this result, increasing the applied current and number of LEDs increases the radiation power. Figure 7 shows the spectral characteristic and dominant frequency of LEDs according to the photoluminescence test. Table 3 shows the means and standard deviations of the measurements for each wavelength in the visible spectrum. The results show that the power of the red and blue wavelengths was dominant in radiometry.

### 3.2. Pulse Measuerment Test

**Figure 8:**
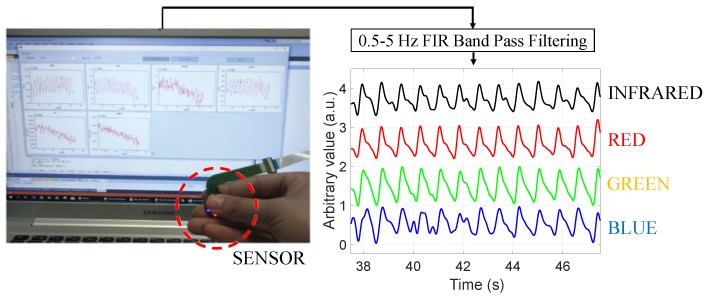
Demonstration of a multi-wavelength PPG measurement while using the proposed omnidirectional optical sensor.

**Figure 9:**
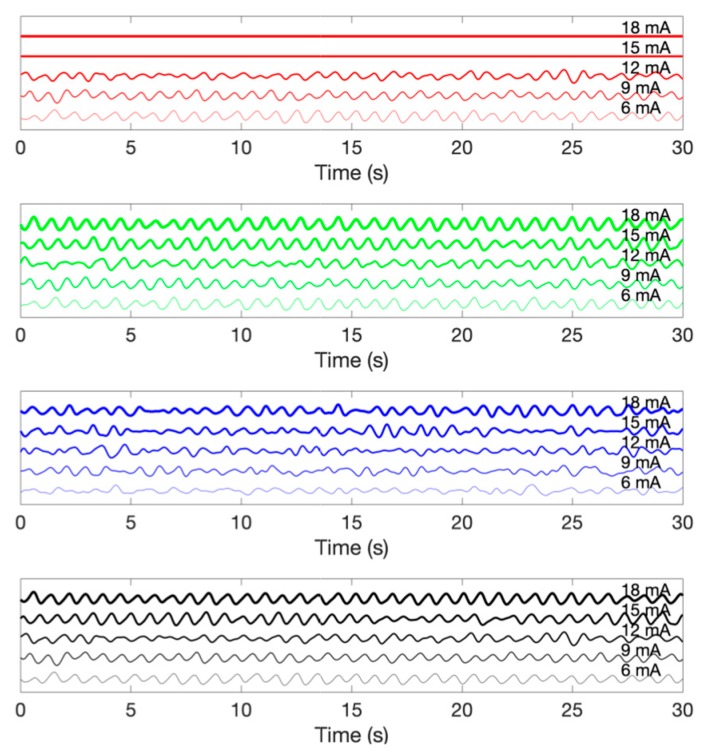
An example of photoplethysmogram according to the driving current. From the top, photoplethysmogram obtained by red, green, blue and infrared wavelength LEDs.

**Figure 10:**
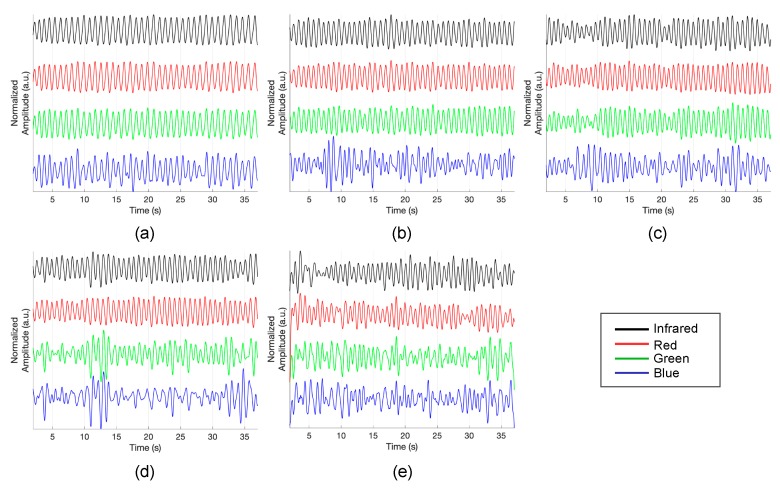
Photoplethysmogram at different measuring sites. (**a**) fingertip, (**b**) earlobe, (**c**) toe, (**d**) wrist and (**e**) forehead.

Figure 8 demonstrates the multi-wavelength PPG measurements by the LEDs in the inner layer. The obtained signal was filtered while using a finite impulse response (FIR) bandpass filter with a 0.5–5 Hz passband. Although there is a difference in signal quality, it can be seen that pulsatile components are observed in the PPG of all wavelengths. Figure 9 shows the PPG waveform that was obtained for each drive current. The measured waveforms were acquired while using four LEDs in the inner layer. In the case of the red wavelength, pulsatile waveforms were observed when driving currents within a range of 6 mA to 12 mA; however, the obtained value is saturated when a current of 15 mA or more was applied, which might be due to the spectral sensitivity of the photodetector. On the other hand, in the green and infrared wavelengths, pulsatile waveforms were observed at all current driving ranges, from 6 mA to 18 mA. In the case of the blue wavelength, the signal quality was generally poor when compared to other wavelengths. However, it can be seen that the pulsatile waveform becomes clearer as the applied current increases. Figure 10 shows the measured PPG waveforms at various measuring sites. In every measuring sites, the pulsatile waveforms are clearly observed, except for the PPG of the blue wavelength. Two reasons can be considered because of poor signal quality at the blue wavelength. First, it is assumed that the sensitivity was lower when acquiring the signal with the blue wavelength, which was the shortest wavelength used, since the sensitivity of the photodetector used in this study decreases as the wavelength gets shorter. A more fundamental reason is the penetration depth, depending on the wavelength used. Previous studies have shown that the blue wavelength penetrates into the vicinity of the epidermis [11]. The PPG waveform appears unstable because the blood vessels are not distributed in the epidermis. In addition, observing the waveforms that were obtained from various measurement points, the waveforms obtained at points closer to the periphery, such as fingertips, toes, and earlobes, showed more stable waveforms than those that were obtained from the wrist and forehead. A pulsatile waveform was not observed by PPG for all wavelengths in signal acquisition from the outer layer of the sensor. This result can be interpreted through the results of Chatterjee’s study [15]. According to their study, the simulation results show that there is only little difference in penetration depth between systolic and diastolic phases when the source-detector distance is approximately 10 mm, and they recommend that the source-detector distance be <6 mm when using multiple wavelengths. If previous studies show the simulation results for the effect of source-detector distance, the results of this study are meaningful for verifying the results based on actual experiments.

**Figure 11:**
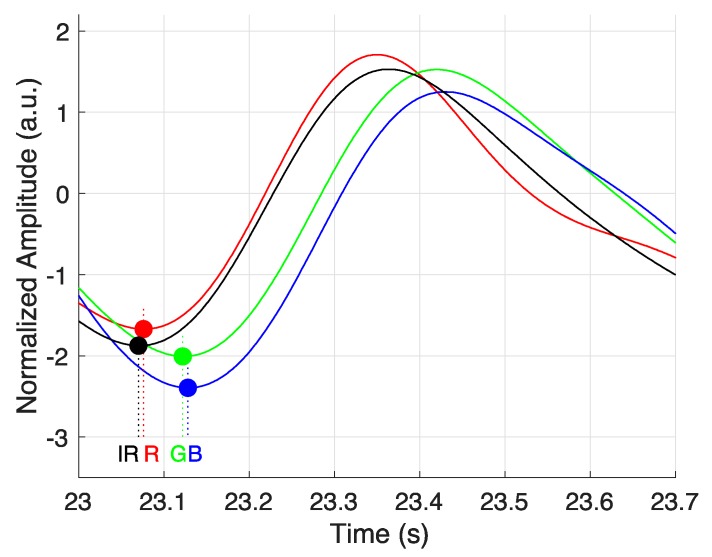
Temporal difference of measured photoplethysmogram in different wavelengths.

In this study, it is assumed that PPG is acquired in different depth of blood vessels according to wavelength. According to this, PPG should be observed first in the deepest blood vessel and it should be observed late in the blood vessel, which is the shallowest. Therefore, the order in which pulses are delivered should be in the IR-RED-GREEN-BLUE order. Figure 11 shows an example of the time difference of PPG that was obtained by wavelength. The circle marker in Figure 11 shows the onset of PPG for each wavelength. As a result, we can confirm that onset occurs in the order of IR-RED-GREEN-BLUE, which is consistent with the hypothesis of the study. 

However, it is necessary to consider that the fluctuation of the transmission depth, depending on the light intensity for each wavelength, can affect the pulse onset point.

## 4. Conclusions

This study suggests a multi-wavelength optical sensor with circular configuration for depth-dependent PPG measurement and evaluates the possibility of PPG measurement. It can be expected that circular placement of multiwavelength optical sensors will become popular when considering that circular sensor placement is already being applied to many wearable devices, including Apple Watch, in single wavelength PPG measurements. In this regard, our research confirms the feasibility of measuring PPG while using a multi-wavelength circular optical sensor and might provide insights into future sensor fabrication. Additionally, it is expected to be applied to various clinical applications, such as hemodynamic analysis or blood pressure estimation of multi-wavelength PPG measurements in the future.

## Figures and Tables

**Table 1:** Specification of the Spectro 320 Scanning Spectrometer.

Property	Value
Spectral range	200−920 nm
Spectral resolution	0.07 nm
Wavelength	± 0.3 nm
VIS sensitivity range for irradiance	2 · 10^−15^ W/nm
VIS signal sensitivity at 1s integration time	1 · 10^−7^ W/m^2^nm
Scan speed	10−100 msec/nm
Intensity	0.01 mcd–800 cd or over
Luminance flux	0.02 mlm–400 lm
VIS, visible spectrum; CRI, color rendering index	

**Table 2:** Radiometric results for each wavelength in the visible spectrum according to the applied current.

Number of LED	Color	Radiant Flux According to the Current (mW)				
		6 mA	9 mA	12 mA	15 mA	18 mA
1	R	0.721 ± 0.081	1.231 ± 0.098	1.733 ± 0.121	2.228 ± 0.153	2.740 ± 0.173
	G	0.376 ± 0.052	0.601 ± 0.074	0.803 ± 0.072	0.991 ± 0.074	1.158 ± 0.083
	B	0.914 ± 0.032	1.439 ± 0.048	1.975 ± 0.036	2.471 ± 0.034	2.910 ± 0.130
2	R	1.393 ± 0.043	2.270 ± 0.076	3.149 ± 0.095	4.059 ± 0.110	4.919 ± 0.124
	G	0.980 ± 0.023	1.422 ± 0.027	1.826 ± 0.039	2.191 ± 0.017	2.524 ± 0.038
	B	1.477 ± 0.041	2.292 ± 0.029	3.060 ± 0.046	3.817 ± 0.020	4.536 ± 0.036
4	R	2.930 ± 0.105	4.653 ± 0.213	6.365 ± 0.295	8.068 ± 0.368	9.815 ± 0.498
	G	1.718 ± 0.024	2.433 ± 0.030	3.086 ± 0.034	3.650 ± 0.030	4.201 ± 0.029
	B	2.961 ± 0.052	4.487 ± 0.046	5.956 ± 0.068	7.372 ± 0.064	8.719 ± 0.068

**Table 3:** Photoluminescence results for each wavelength in the visible spectrum.

Property/Color	Red	Green	Blue
Peak wavelength (nm)	660.8 ± 1.0	528.6 ± 24.5	448.0 ± 0.4
Centroid wavelength (nm)	653.5 ± 3.8	513.0 ± 1.6	432.8 ± 15.2
Dominant wavelength (nm)	644.4 ± 3.2	538.2 ± 1.2	453.5 ± 0.5
